# Supplementary material for: Local disorder in Na0.5Bi0.5TiO3-piezoceramic determined by 3D electron diffuse scattering
Source: Sci Rep. 2017 Oct 2;7:12519. doi: 10.1038/s41598-017-12801-w (PMC5624983; doi:10.1038/s41598-017-12801-w)
Supplement: Supplementary file 1 — Supplementary information [file 41598_2017_12801_MOESM1_ESM.pdf]

Local disorder in $Na_{0.5}Bi_{0.5}TiO_3$-piezoceramic determined by 3D electron diffuse scattering

Alexandra Neagu[1] and Cheuk-Wai Tai[1]

[1]Department of Materials and Environmental Chemistry, Stockholm University, SE-106 91 Stockholm, Sweden

Corresponding authors – email addresses:
Alexandra Neagu: alm.neagu@gmail.com
Cheuk-Wai Tai: cheuk-wai.tai@mmk.su.se

Supplementary information

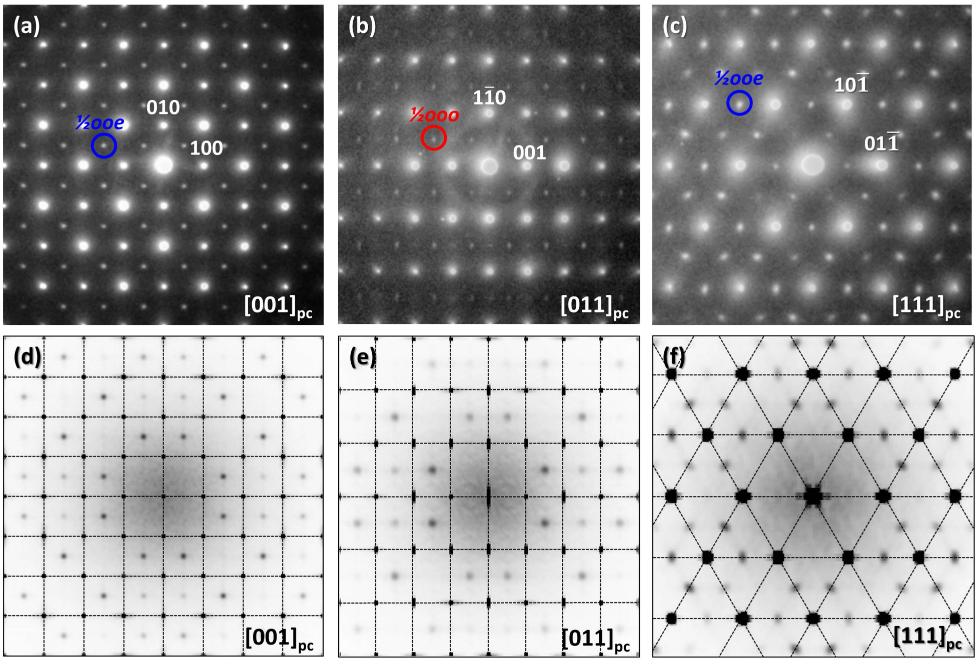

Supplementary Figure S1. Experimental SAED patterns and kinematical simulated electron diffraction patterns for 85NBT-10BKT-5BT ternary compound. The experimental SAED patterns have been recorded along **a.** $[001]_{pc}$, **b.** $[011]_{pc}$ and **c.** $[111]_{pc}$ zone axes. The simulated electron diffraction patterns were calculated along the same zone axes, namely **d.** $[001]_{pc}$, **e.** $[011]_{pc}$ and **f.** $[111]_{pc}$. For simplicity and convenience of comparison pseudocubic axes ($Pm\bar{3}m$) have been used for indexing.

Two types of superstructure reflections with respect to the ideal cubic perovskite structure can be observed, namely ½(*ooe*) as depicted by blue circles in ZAs $[001]_{pc}$ and $[111]_{pc}$ and ½(*ooo*) as depicted by the red circle in ZA $[011]_{pc}$. All patterns have been calculated from a 50×50×50 supercell with initial in-phase tilted domains of 10×10×2 unit cells large and a 30/70 antiphase/in-phase tilting ratio. At this stage no cation displacements or short range chemical order has been considered. The kinematical approximation was used for calculating the electron diffraction patterns. In the case of experimental SAED patterns, kinematically forbidden reflections often appear due to the occurrence of double diffraction. For example the $a^0a^0c^+$ tilt system allows for superstructure reflections that have $h \neq \pm k$. Hence, the *1/2 1/2 0* reflection in ZA $[001]_{pc}$ is kinematically forbidden but appears due to double diffraction, such as the route *3/2 3/2 0* ± 1-10. In the case of $a^-a^-a^-$ tilt system the condition for allowed superstructure reflections is $h \neq \pm k$, $k \neq \pm l$ and $l \neq \pm h$ and similar to the *1/2 1/2 0* reflection the *1/2 1/2 1/2* reflection in ZA $[011]_{pc}$ is also forbidden and appears due to double diffraction. Taking into consideration double diffraction and the fact that the kinematical approximation

was used for calculating the simulated electron diffraction patterns, a good agreement was obtained between the experimental patterns and the simulated ones. All superstructure reflections have been accounted for with a two-tilt system model which includes the $a^0a^0c^+$ and $a^-a^-a^-$ tilt systems.

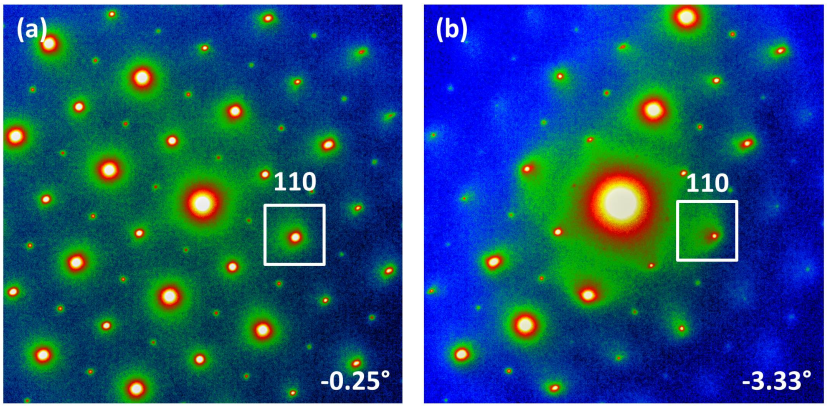

Supplementary Figure S2. Experimental SAED patterns. a. Electron diffraction pattern very close to $[001]_{pc}$ ZA. **b.** Electron diffraction pattern ~3° away from $[001]_{pc}$ ZA. Broad diffuse scattering intensity near the fundamental perovskite reflections can be clearly observed in (b) as highlighted by the white square for 110 reflection. However this is not the case for the electron diffraction pattern (a) which is very close to exact ZA condition.

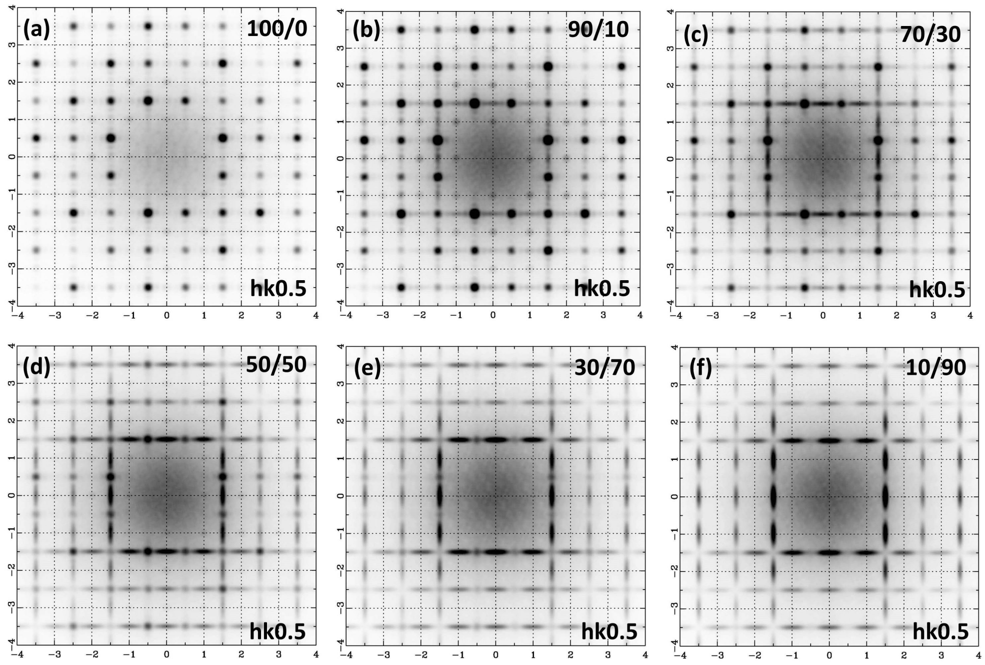

Supplementary Figure S3. Simulated electron diffraction patterns in the reciprocal-space plane *hk0.5* with different antiphase/in-phase tilting ratios. All patterns have been calculated from a 50×50×50 supercell with tetragonal domains of 10×10×2 unit cells large but with different antiphase/in-phase ratios: **a.** 100/0, **b.** 90/10, **c.** 70/30, **d.** 50/50, **e.** 30/70 and **f.** 10/90. Electron diffraction patterns have been calculated using the kinematical approximation and by averaging the results from 20 different simulations, in order to reduce the anisotropic contribution to the diffraction intensities.

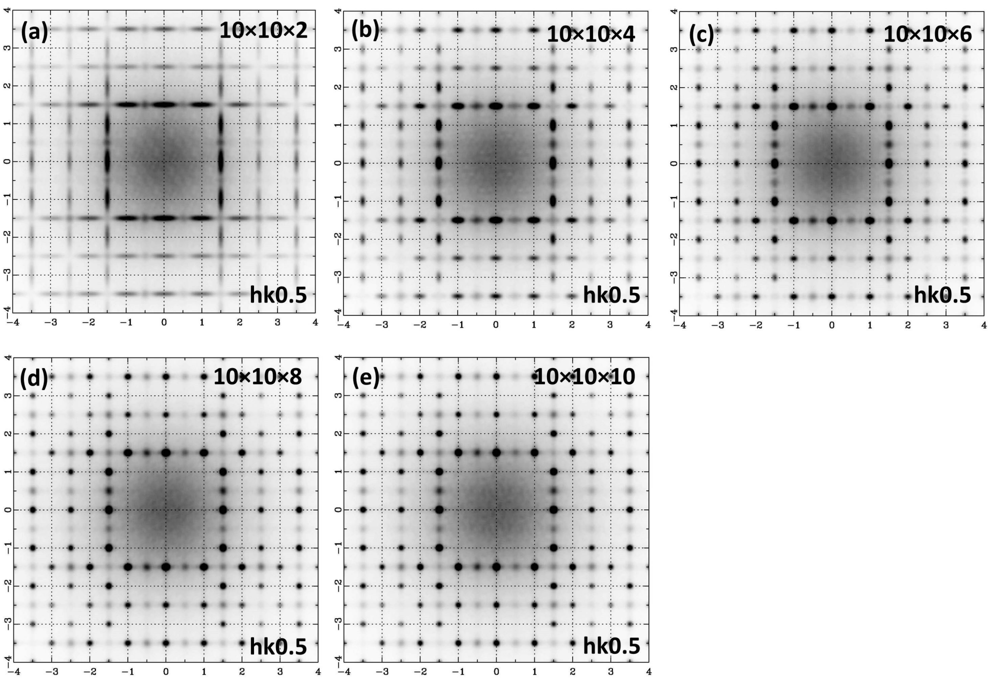

Supplementary Figure S4. Simulated electron diffraction in the reciprocal-space plane *hk0.5* with different shapes for the in-phase domains. All patterns have been calculated from a 50×50×50 supercell with an antiphase/in-phase ratio of 30/70 but using different shapes for the in-phase domains: **a.** 10×10×2 unit cells **b.** 10×10×4 unit cells **c.** 10×10×6 unit cells, **d.** 10×10×8 and **e.** 10×10×10 unit cells. Electron diffraction patterns have been calculated using the kinematical approximation and by averaging the results from 20 different simulations.
